# Persian chin holder and retractor

**DOI:** 10.1186/s40902-023-00376-8

**Published:** 2023-02-17

**Authors:** Seied Omid Keyhan, Rata Rokhshad

**Affiliations:** 1 Department of Oral & Maxillofacial Surgery, College of Dentistry, Gangneung-Wonju National University, Gangneung, South Korea; 2 Department of Oral & Maxillofacial Surgery, College of Medicine, University of Florida, Jacksonville, FL USA; 3 Maxillofacial Surgery & Implantology & Biomaterial Research Foundation, Isfahan, Iran; 4 Iface Academy, Atlanta, GA USA; 5 Topic Group Dental Diagnostics and Digital Dentistry, ITU/WHO Focus Group AI on Health, Berlin, Germany; 6 Section of Endocrinology, Nutrition, and Diabetes, Department of Medicine, Boston University Medical Center, Boston, MA USA

Genioplasty can be challenging due to the difficulty of maintaining adequate tension on organs and other structures. Because the chin projects and is vertically long, it contributes to facial esthetic harmony in the lower third of the face. Skeletal and soft tissue appearance can be radically altered by changing these supports. Minimally invasive surgery causes fewer complications, requires fewer analgesics, can be performed in a shorter period, and is associated with a faster recovery time and an earlier return to normal activities [1].

An older system relies on screws or wires. Stainless steel retractors protect nerves and tissues at the lower edge of the chin. An incision or wound is held open with a surgical retractor while a surgeon works. As well as holding tissues and organs out of the way during surgery, a retractor can be used to keep them out of harmful way. In addition to musculoskeletal disorders, manual retraction needs more operators and more time [2]. A surgical self-retaining retractor (SSR) is a device that allows perioperative care providers to decide when and under what circumstances it is safe to retract tissue manually. Benefit of SSR includes freeing up an assistant’s hands during surgery. The surgical site can now be accessed more easily by SSR. Adjustments can be made with one hand. Incisions are less cluttered with instruments. New types of SSR that are lighter and easier to use are available. Operation can be done by fewer staff. For novices, this instrument will be an excellent visual aid. The reduced infection risk in an operating theater/operating room has been expected because fewer staff are present [3, 4]. Instead of assistants leaning in close in often awkward positions to assist for long periods of time, SSR can be used. It is cost benefit as the following happens: retraction is not required by the assistant, making surgery faster, decreasing staff costs, and improving workplace ergonomics. Moreover, local injury can be prevented by blunt-edged profiles.

Mandibular reconstructive procedures are usually performed with it to assist in maintaining an optimal chin position. The protuberance of the mental cortex is supported by four holes located on the blade, which can be fixed to chin using 1.8 mm screws for further performances during surgeries, such as moving the segment anteriorly, laterally, or posteriorly (Figs. 1 and 2). The main disadvantage of using wires is their disability for moving segments laterally or posteriorly [5]. The blade is deeply curved for this purpose. A curve can also assist in retracting the lower lip, thereby revealing the anterior mandible body. It is rigidity, no interference with surgery site, and after fixation, it helps the accessibility, visibility, and stability for fixing screws and procedure, especially in orthognathic surgeries for prognathic patients and achieving symmetrical mandibular. By using this new instrument, surgeons are able to obtain an optimal surgical field, which is extremely useful for genioplasty surgery.

**Fig. 1:**
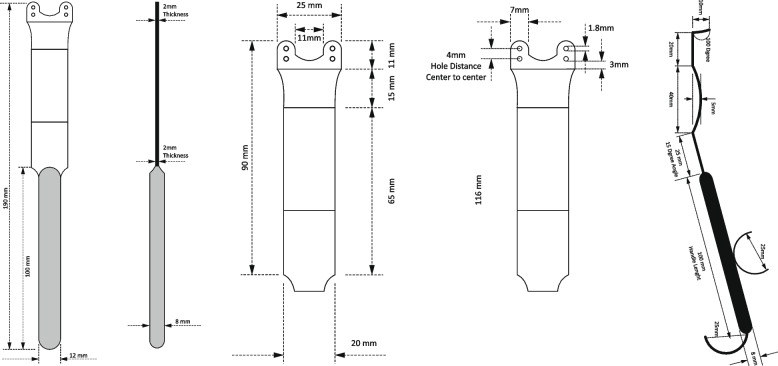
Dimensions of the Persian chin holder and retractor

**Fig. 2:**
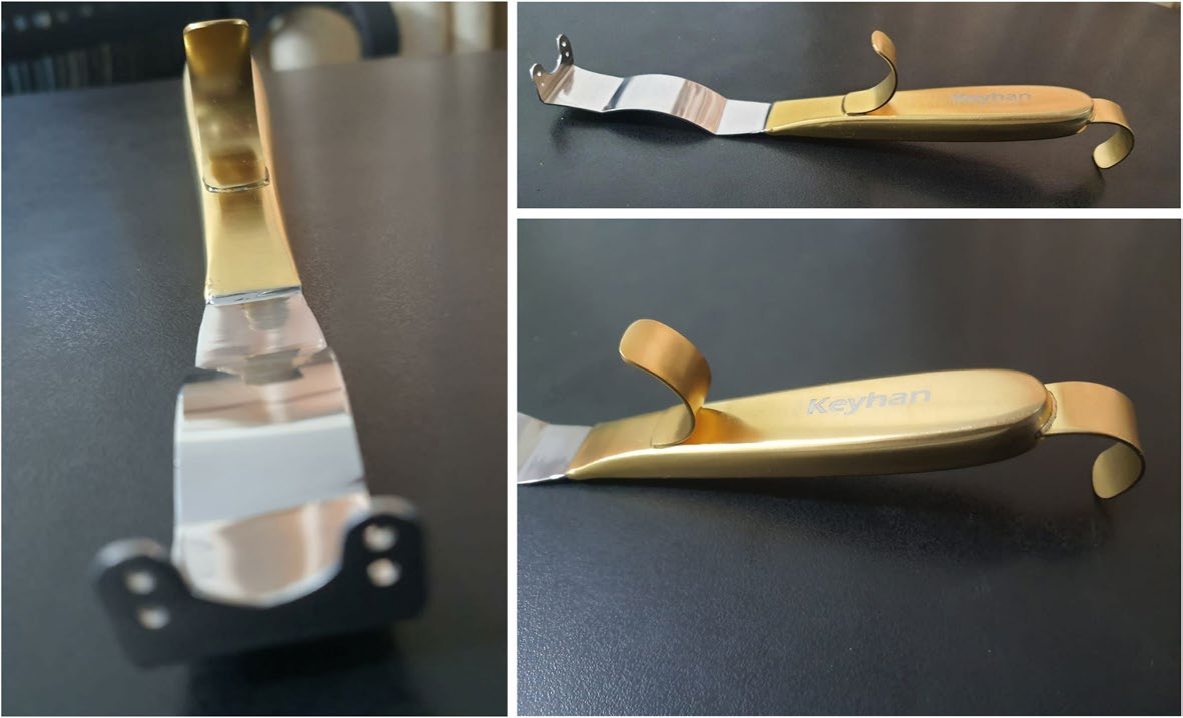
Different views of Persian chin holder & retractor

## Data Availability

Not applicable.
